# Measures for Combatting Sterility

**Published:** 1851-08

**Authors:** 


*Measures for Combating Sterility.*—We find that Dr. Mistler has published in the *Gazette Médicale de Strasbourg* a method of his, for rendering barren women fertile. the author thinks that an abnormal narrowness of the neck and mouth of the uterus is the principal cause of sterility, and first cauterizes the internal portion of the neck with nitrate of silver, and then proceeds to dilate that canal with cones of prepared sponge, the size of which is gradually increased. Nine women were thus treated, seven of whom bore children after the above means had been persevered in for several months.—*Ibid.*

